# Walking is the form of physical activity people with osteoarthritis in the hip or knee choose and maintain after two years

**DOI:** 10.1016/j.ocarto.2025.100681

**Published:** 2025-09-11

**Authors:** R. Bendrik, M. Peterson, K. Bröms, M. Emtner, A. Hed Ekman, L.V. Kallings, B. Sundström

**Affiliations:** a Department of Public Health and Caring Sciences, General Practice, Uppsala University, Uppsala, Sweden; b Centre for Research and Development, Uppsala University/Region Gävleborg, Gävle, Sweden; c Academic Primary Health Care, Region Uppsala, Sweden; d Department of Medical Sciences, Respiratory-, Allergy and Sleep Research, Uppsala University, Uppsala, Sweden; e Department of Occupational Health Science and Psychology, University of Gävle, Gävle, Sweden; f Department of Physical Activity and Health, Swedish School of Sport and Health Sciences, GIH, Stockholm, Sweden

**Keywords:** Osteoarthritis, Physical activity, Walking, Counselling, Patient-centred

## Abstract

**Objective:**

To evaluate which form and type of physical activity individuals with hip or knee osteoarthritis choose and maintain one and two years after an individualised intervention for physical activity and further to evaluate whether there were differences in the most chosen physical activity with regard to patient characteristics.

**Method:**

Patients with hip or knee osteoarthritis from a previous randomised controlled trial, where they received individualised patient-centred counselling about physical activity and registered self-selected sessions of physical activity in a 7-day diary, were included. Sessions lasting more than 10 ​min and rated at least light effort were categorized, and differences were evaluated.

**Results:**

Of the 94 patients included, 72.3 ​% were female, 72.0 ​% had knee osteoarthritis and mean age was 62.0 (SD 8.2) years. Women and men who preferred walking, walked on average ​> ​4 times/week and 3–4 times/week, respectively. Everyday activities and cycling were performed 2–3 times/week by both women and men. The proportions of individuals maintaining the same activity after one and two years were 50 ​% for walking and 2 ​% for strength training. Men more often choose different activities and after two years they performed everyday activities and cycling to the same extent as walking. Individuals who chose walking were significantly older, of female gender and had lower muscle strength in the affected leg.

**Conclusion:**

Walking is the form of physical activity patients with osteoarthritis most often choose, perform and maintain. Knowledge about preferred activities among patients is crucial for maintaining physical activity in the long term.

## 1 Introduction

People with osteoarthritis (OA) in the hip or knee often reduce their physical activity [1], despite the potential to achieve pain relief, symptom control, improved quality of life and reduced risk of comorbidity [2,3]. Extensive research has attempted to identify the best exercise treatment and the best way to deliver the treatment [[4], [5], [6], [7]]. Various interventions show a short-term effect in reduced pain and improved function and quality of life [2,3], but no physical activity intervention appears to be better in maintaining physical activity in the long term [8,9]. Therefore, OA guidelines recommend an individualised approach with physical activity interventions based on the individual's needs and preferences [[10], [11], [12]]. Counselling should be patient-centred, meaning that treatment decisions should consider what will work best for the individual according to preferences, access, affordability and medical status [10,13].

Knowledge about which physical activity works for patients in the long term is important in patient-centred counselling, but to our knowledge, few studies have evaluated which form and type of physical activity people with hip or knee OA maintain in the long term when they choose the activity themselves.

The primary aim of this study was to evaluate the form and type of physical activity that people with hip or knee OA perform before and one and two years after an individualised physical activity intervention. A secondary aim was to evaluate whether there were differences in patient characteristics and gender with regard to which physical activity was most frequently chosen by patients.

## 2 Method

### 2.1 Design and patients

This follow-up study used data from a previous randomised controlled trial (RCT) in primary care, which focused on the effect of individualised physical activity on prescription compared to the effect of individualised advice on physical activity counselling. The RCT included patients with clinically verified OA in the hip or knee assessed according to risk factors, symptoms and findings, aged 40–74 years, maintaining less than 150 ​min of moderate physical activity a week [14,15]. The RCT participants were assessed using questionnaires, performance-based tests and x-ray examinations of the affected joint. A full description of the RCT is available in previous articles [14,15]. To be included in the present study, patients must have completed a 7-day physical activity diary in the original RCT at baseline and one and two years after baseline. The original RCT was registered at ClinicalTrial.gov (NCT02387034) and were approved by the Regional Ethical Review Board in Uppsala (Ref. no. 2010/001).

### 2.2 Intervention

The interventions in the RCT from which data about the patients were collected were characterised as “individualised physical activity on prescription” versus “individualised advice about physical activity” [14]. The prescription group was instructed to choose their own form of physical activity. The chosen form and the dosage were discussed with the physiotherapist and was documented on the prescription. Four follow-ups were included during the following six months. The patient and the physiotherapist together evaluated the planned physical activity. Sometimes a new goal was set and a new prescription was made. The advice group had a 1-h session with a physiotherapist. They were instructed to choose their own form of physical activity and to perform that activity for 30 ​min three times per week. In addition they were advised to perform muscle strength activities in daily life, such as climbing stairs. The analysis pooled the data from the previously randomised treatment arms of the RCT into a single group and analysed the pooled data from patients' physical activity diaries. The intervention was summarised as individualised patient-centred counselling based on the patients’ needs and preferences and supporting activities preferred by the patients.

Thus, the patients chose their own activities. The activities could be categorized as aerobic, muscle strengthening, mind-body or mixed activities. The activities could be performed at home, in the surrounding environment or as a supervised activity, depending on the patient's preferences and what they themselves had arranged. The patients participated in one to five physiotherapy sessions over six months.

### 2.3 Outcome - forms of physical activity

The activities performed by the patients were to be recorded in a diary for seven consecutive days at baseline and at one and two years. Walking outdoors, walking on a treadmill and Nordic walking were all defined as a form of *walking*. Gardening, shovelling snow, home cleaning, house painting, washing the car, doing carpentry, chopping firewood and shopping were all defined as *everyday activities*. Outdoor and indoor cycling were defined as *cycling*. Gym training and strength training were defined as *strength training*. Swimming and water training were defined as *water activities*. Yoga and Qigong were defined as *mind-body activities*. Jogging, skiing, skating, dancing, Zumba, riding, bowling, boule and curling were rarely recorded activities and thus were defined as *other activities* (Supplement 1).

The different forms of physical activity the patients performed were evaluated. First, the number of valid sessions per week, defined as a session lasting a minimum of 10 ​min at an exertion of at least 11 on the Borg RPE 6–20 scale [16] was evaluated. We chose these parameters because in patients with hip or knee osteoarthritis there is still limited evidence regarding the optimal intensity and dosage of exercise needed for clinical benefits [10,13]. It appears that low-intensity physical activity [[4], [5], [6], [7]] and a small dose [34] may benefit these patients. Second, the number of patients who performed the same activity both at baseline and after 1 year and 2 years was evaluated.

### 2.4 Outcome - type of physical activity

The seven different forms of physical activity described above were then grouped into four types of physical activity: *aerobic exercise, muscle strengthening, mind-body* [6,7] and *mixed activities*. Walking, treadmill, Nordic walking, jogging, outdoor cycling, indoor cycling, dancing, Zumba, swimming, water activities, skiing, skating, riding, boule, bowling and curling were included in *aerobic exercise*. Gym and strength training were included in *muscle strengthening*. Yoga and Qigong were included in *mind-body*. *Mixed activities* were added as a separate category consisting of a mix of both aerobic exercise and muscle strengthening. Gardening, shovelling snow, house cleaning, house painting, washing the car, carpentry, chopping firewood and shopping were all included in *mixed activities*.

### 2.5 Outcome- gender differences and patient characteristics

We evaluated differences in the most chosen forms of physical activity with regard to patient characteristics. Patient characteristics recorded from baseline were age, gender, body mass index, location of affected leg and severity of OA as assessed by x-ray [17]. Patient characteristics recorded after two years were reported pain (HOOS/KOOS) [18,19], 6-min walk distance [20], reported pain after walking (VAS) [21], muscle strength assessed with the 30-s chair-stand test [20] and maximal step-up test [22].

### 2.6 Statistical analysis

Sessions were sorted by form and type of physical activity then summarised, organised and described in frequencies and percentages. The differences in characteristics between groups were assessed with chi-square or Fisher's test in categorical variables, and with the independent two-tailed test or Mann Whitney test in numerical data. A two-sided *P*-value of less than 0.05 was set for statistical significance. All analyses were performed with the use of SPSS Statistics, version 22 (SPSS Inc., Chicago, IL, USA).

## 3 Results

Ninety-four primary care patients with hip or knee OA completed the 7-day diary at baseline, after one year and after two years and were included in the present study. Forty-seven patients from the original RCT were not included because they lacked completed diaries (n ​= ​12) or dropped out of the original study (n ​= ​35). There were no differences between included and excluded patients regarding age or BMI, but more severe OA, defined as a Kellgren-Lawrence score 3–4 [17], was more common among the excluded patients (30 ​% vs 11 ​%, p ​< ​0.001). The characteristics of the included patients are presented in Table 1.

**Table 1:** Participant characteristics of 94 patients with hip or knee osteoarthritis participating in an individualised physical activity intervention.

Characteristics	n ​= ​94
Age, mean (SD)	62.0 (8.2)
Women, n (%)	68 (72)
BMI, mean (SD)	30.2 (4.4)
Radiographic Kellgren-Lawrence score 3–4^a^, n (%)	10 (11)
Location OA, knee, n (%)	68 (72)
Hip	26 (28)
Comorbidity	
Heart disease^b^, n (%)	19 (20)
Severe obesity, (body mass index (kg/m^2^) >35, n (%)	12 (13)
Asthma/COPD, n (%)	10 (11)
Depression, n (%)	6 (6)
Severe pain (not due to knee or hip, n (%)	5 (5)
Diabetes mellitus, n (%)	5 (5)
HOOS/KOOS, mean (SD)	
Pain	53 (17)
Symptom	57 (18)
Activities of daily living	62 (18)
Sports and recreation	31 (23)
Quality of life	39 (17)

Abbreviations: SD ​= ​standard deviation, BMI ​= ​body mass index, COPD ​= ​chronic obstructive pulmonary disease, HOOS= Hip disability and Osteoarthritis Outcome Score, ranges from 100 (best) to zero (worst), KOOS=Knee injury and Osteoarthritis Outcome Score, ranges from 100 (best) to zero (worst).

a Scores on the Kellgren-Lawrence scale range from 1 to 4, higher scores indicate more severe disease.

b Heart disease: myocardial infarction, angina pectoris or heart failure.

### 3.1 Forms of physical activity

**Fig. 1:**
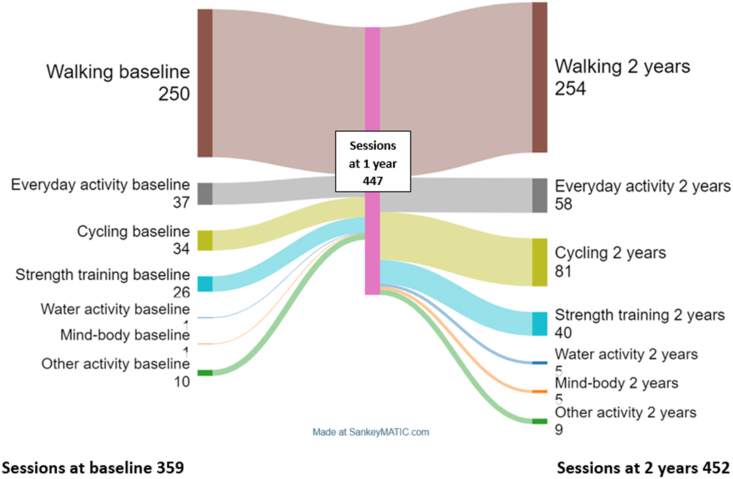
Sankey diagram illustrating changes in the number of weekly sessions across different types of physical activity among 94 patients with hip or knee osteoarthritis. Each flow represents patients transitioning between different activities over time, with wider flows indicating larger groups.

Among the 94 patients included, walking was the most frequent form of physical activity at baseline. At the baseline assessment, patients who walked performed 4.2 sessions of walking per week, those who chose *everyday activities* 1.8 sessions per week and those who chose cycling 2.6 sessions per week (Table 2). After two years, walking was still the most common activity both in terms of number of sessions performed and the number of patients who walked (Table 2). Walking was the form of physical activity that 50 ​% of the individuals maintained at all three time points. Of these 47 patients, 12 reported that another physical activity was also performed at all three time points (everyday activity (5), cycling (4), strength training (2) and other activity/boule (1). Everyday activities were maintained by 13 ​% of the patients at all three time points (Table 2). The activity that increased the most from baseline to two years after baseline in both frequency and number of patients was cycling (Fig. 1). All registered activity sessions are presented in detail in Supplement 1.

**Table 2:** Forms of physical activity and number of patients with hip or knee osteoarthritis participating in an individualised intervention for physical activity – at baseline and after 1 and 2 years.

	Baseline		1 year		2 year		Same activity all three times
	sessions	patients n ​= ​94	sessions	patients n ​= ​94	sessions	patients n ​= ​94	patients n ​= ​94
Walking^a^	250	60	286	63	254	62	47 (50 ​%)
Everyday activities^b^	37	21	54	25	58	31	12 (13 ​%)
Cycling^c^	34	13	56	22	81	26	5 (5 ​%)
Strength training^d^	26	14	28	16	40	18	2 (2 ​%)
Water-activities^e^	1	1	7	4	5	4	0 (0 ​%)
Mind-body^f^	1	1	4	3	5	4	0 (0 ​%)
Other activities^g^	10	9	12	8	9	7	1 (1 ​%)
No-activity	0	23	0	14	0	10	4 (4 ​%)
Total number of sessions	359		447		452		

a Walking ​= ​walking, Nordic-walking, treadmill walking.

b Everyday activities ​= ​gardening, shovelling snow, cleaning the house, painting the house, washing the car, carpentry, chopping firewood, shopping.

c Cycling = outdoor and indoor cycling.

d Strength training ​= ​gym-training, strength training.

e Water activities ​= ​swimming, water training.

f Mind-body ​= ​yoga, Qigong.

g Other activities ​= ​jogging, skiing, skating, dance, Zumba, riding, boule, bowling, curling.

### 3.2 Type of physical activity

**Fig. 2:**
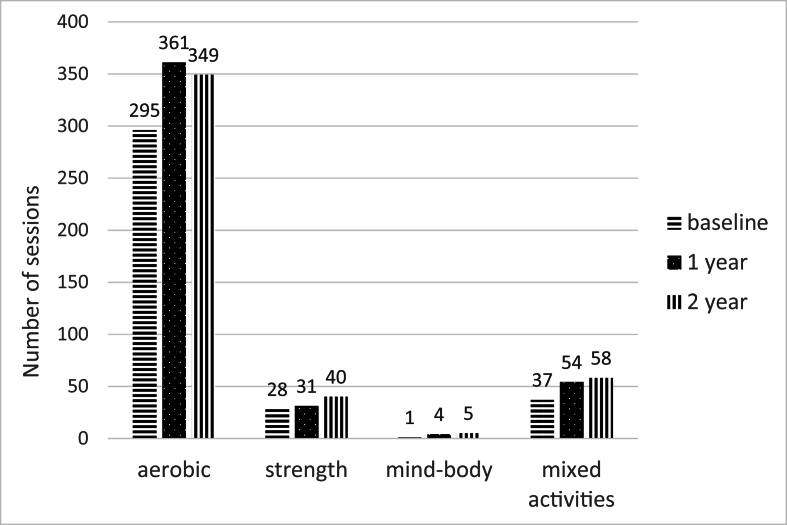
Number of sessions per week in different types of physical activity recorded by 94 patients with hip or knee osteoarthritis participating in an individualised intervention for physical activity, at baseline and after one and two years. Aerobic ​= ​walking, treadmill, Nordic walking, jogging, outdoor cycling, indoor cycling, dancing, Zumba, swimming, water activities, skiing, skating, riding, boule, bowling, curling. Muscle strength ​= ​gym, strength training. Mind-body ​= ​yoga, Qigong. Mixed activities ​= ​gardening, shovelling snow, house cleaning, house painting, washing the car, carpentry, chopping firewood, going shopping.

Of the categories *aerobic exercise, muscle strengthening, mind-body* [6,7] and *mixed activities,* aerobic exercise was the most prominent type of physical activity at all three time points (Fig. 2).

### 3.3 Gender differences and patient characteristics

Overall, 73.5 ​% of the women walked, both at baseline and after two years, while 38.5 ​% of men walked at baseline and 46.2 ​% at two years. Men also performed everyday activities to the same extent as walking, and cycling increased from 7.7 ​% to 42.3 ​% at two years. The form of activity most frequently performed by both women and men at all three time points was walking (Table 3).

**Table 3:** Number of sessions and number of women and men with hip or knee osteoarthritis in the four most common forms of physical activity, in individuals participating in an individualised physical activity intervention, reported at baseline and after one and two years.

Form of activity	Overall n ​= ​94		Women n ​= ​68		Men n ​= ​26		*p*-value^e^
	sessions	n (%)	Sessions	n (%)	sessions	n (%)	
**Walkin**g^a^							
Baseline	250	60 (63.8)	217	50 (73.5)	33	10 (38.5)	
1 year	286	63 (67.0)	238	52 (76.5)	48	11 (42.3)	
2 year	254	62 (66.0)	217	50 (73.5)	37	12 (46.2)	**0.012**
**Everyday activity**^b^							
Baseline	37	21 (22.3)	17	11 (16.2)	20	10 (38.5)	
1 year	54	25 (26.6)	33	16 (23.5)	21	9 (34.6)	
2 year	68	31 (33.0)	37	18 (26.5)	31	13 (50.0)	**0.020**
**Cycling**^c^							
Baseline	34	13 (13.8)	28	11 (16.2)	6	2 (7.7)	
1 year	56	22 (23.4)	43	16 (23.5)	13	6 (23.1)	
2 year	81	26 (27.7)	47	15 (22.1)	34	11 (42.3)	0.050
**Strength training**^d^							
Baseline	26	14 (14.9)	22	11 (16.2)	4	3 (11.5)	
1 year	28	16 (17.0)	26	15 (22.1)	2	1 (3.8)	
2 year	40	18 (19.1)	28	13 (19.1)	12	5 (19.2)	0.597

a Walking ​= ​walking, Nordic-walking, treadmill walking.

b Everyday activities ​= ​gardening, shovelling snow, cleaning the house, painting the house, washing the car, carpentry, chopping firewood, going shopping.

c Cycling ​= ​outdoor and indoor cycling.

d Strength training ​= ​gym-training, strength training.

e p-value ​= ​between groups, compares the number of women and men at 2 years in the different forms of activity.

Those who preferred walking were older (p ​< ​0.001) and more often female (p ​= ​0.012). We also found that individuals preferring walking had significantly lower muscle strength (p ​= ​0.010) compared to non-walkers, measured as maximal step-up height in the affected leg. No difference was seen between walkers and non-walkers with regard to BMI, location of the affected joint, grade of OA, reported pain, walking distance and performance in the 30-s chair-stand test (measure of muscle strength in both legs) (Table 4).

**Table 4:** Characteristics of walkers and non-walkers of 94 patients with hip or knee osteoarthritis participating in an individualised physical activity intervention, compared at baseline (age, gender, body mass index, affected joint, radiographic severity) and after 2 years (function, pain).

	All (n ​= ​94)	Walkers (n ​= ​62)	Non-walkers (n ​= ​32)	Between-group *P*-value
Age, mean (SD)	62.0 (8.2)	64.1 (7.6)	57.9 (7.7)	**<0.001**
Women, n (%)	68 (72.3)	50 (73.5)	18 (26.5)	**0.012**
BMI, mean (SD)	30.2 (4.4)	30.2 (4.4)	30.3 (4.5)	0.891
Affected joint/knee OA, n (%)	68 (72.3)	43 (69.4)	25 (78.1)	0.468
Radiographic OA severity^a^, grade 3–4, n (%)	10 (10.6)	8 (12.9)	2 (6.3)	0.519
30-s chair-stand test, n, mean (SD)	13.5 (3.7)	13.0 (3.5)	14.4 (3.9)	0.095
Maximal step-up height affected leg, cm, mean (SD)	26.9 (6.7)	25.6 (6.2)	29.5 (6.8)	**0.010**
Six-minute walk distance, meter, mean (SD)	531 (96)	520 (94)	555 (98)	0.113
Pain (VAS) after walking 6-min, (IQR)	5.0 (25.0)	5.5 (30.3)	3.5 (24.0)	0.529
Pain (HOOS/KOOS), 0-100, median (IQR)	63.9 (36.1)	68.7 (35.0)	63.0 (36.9)	0.389

Abbreviations: SD ​= ​standard deviation. OA ​= ​osteoarthritis. BMI ​= ​body mass index. VAS ​= ​visual analogue scale, ranges from zero (best) to 100 (worst). HOOS= Hip disability and Osteoarthritis Outcome Score, ranges from 100 (best) to zero (worst). KOOS=Knee injury and Osteoarthritis Outcome Score, ranges from 100 (best) to zero (worst).

a Scores on the Kellgren-Lawrence scale range from 0 to 4, higher scores indicate more severe disease.

## 4 Discussion

In this study, we evaluated which form and type of physical activity patients with hip or knee OA prefer to do in the long term, when they choose the physical activity themselves. The results clearly show that walking was the most frequent physical activity performed per week by both women and men and the activity they most often maintained in the long term. More than 73 ​% of women preferred walking, both at baseline and after two years. Men preferred a mix of activities, such as walking, everyday activities and cycling. Individuals who preferred walking were older, more likely to be female and had lower muscle strength in the affected leg compared to non-walkers. With regard to the type of activity, aerobic physical activity was the most popular, while few patients preferred muscle strengthening and mind/body activities.

Long term adherence to exercise is generally poor and very few studies examine and characterize what form of physical activity people with OA prefer and maintain. This study adds new qualitative and quantitative long term data about physical activity in people with OA of the hip and knee. In a cross-sectional design, Castañeda et al. found that walking was the most commonly reported form when people with hip or knee osteoarthritis choose their own activities. However, they found no difference between women and men regarding walking [23]. In a previous cohort study by Lo et al., people with knee OA were asked specific questions about walking, which showed, as in our study, that walking was a popular form of physical activity. In that study, 73 ​% reported walking as their exercise of choice [24]. The authors also found significant long term benefits for those who walked compared to non-walkers [24]. After 48 months, the walking group had less knee pain and better x-ray status compared to the non-walking group [24]. Similar to our study, differences were found between women and men in a systematic review that evaluated general populations (not OA patients) [25]. Here, the authors found that women reported a higher prevalence of walking than men, and the findings were largely consistent across countries [25]. In our study, self-selected walking appears to be an activity that patients maintain for a long time, and this is in line with other studies that have compared self-selected walking with supervised walking and found benefits for both self-selected and supervised walking at 18 months [26] and at 12 months [27].

Our results confirm that walking seems to be a form of physical activity that suits the majority of patients with OA, regardless of pain level, BMI and the severity of OA. This is in agreement with other studies showing that participation in physical activity is not related to the perceived level of pain [28] nor BMI [29], and that the treatment effect is similar, irrespective of the baseline radiographic OA severity [30]. We found that individuals preferring walking were on average significantly weaker in the affected leg and older compared to those preferring other forms of activity. A simple explanation may be that walking is easy to do and maintain in the long term, and that it can therefore suit physically weaker and older individuals.

The finding that walking and everyday activity were forms of physical activity the patients themselves maintained in the long term raises the question of whether the focus of counselling on physical activity should be changed for people with hip or knee OA. Extensive research has evaluated the effectiveness of specific exercises [[4], [5], [6], [7]] and how to maintain adherence to these activities [31,32]. We believe that a better counselling strategy could be to support activities that the patient already does or is accustomed to doing. Counselling should be offered at the early stages of OA, with a focus on what the individual believes can work in the long term. Offering support that allows individuals to choose activities themselves improves the patient's confidence and empowerment [33]. Perhaps patient empowerment could be further increased if, for example, patients who choose to walk can be informed that even a small amount of walking, as little as 70 ​min per week, can have benefits for cardiovascular health [34]. If physical activity counselling is a continuous process, it is possible to support the patient to maintain self-selected activities and, if necessary, individually discuss how to add other activities such as strength training or discuss how intensity can be increased.

In our study, few patients performed and maintained strength training. Only 2 ​% of patients completed strength training at all three time points. This is an important finding that should be evaluated further because these patients are at risk of decreasing muscle strength [35]. Further studies are warranted to identify which interventions help patients with OA to maintain and increase muscle strengthening activities in the long term. In a previous study, we found that simply advising patients to perform muscle strengthening activities in daily life (climbing stairs and focusing on the legs when rising from a chair) improved leg muscle strength to the same extent as a more comprehensive intervention when evaluated at 12 and 24 months [15]. Given that such a minor intervention seems to improve muscle strength, this should be investigated in further studies.

The strength of the study was that we described a wide range of physical activities that people with hip or knee OA selected themselves. Recall bias was minimised as patients were asked to record the performed activities in the diary the same day. Since we evaluated the patients during the same season at all three time points, we eliminated the influence of seasonal differences. A limitation of our study may be that we only evaluated the frequency of sessions and not the total time of physical activity performed in every session. However, we believe that a description of the number of sessions per week within different physical activities adequately describes the physical activity pattern. It is important to know which activities work long term for these patients as guidelines emphasize shared decision-making between caregivers and patients, and that patient's values and preferences should be considered [[10], [11], [12]]. Another limitation is that we included activities that patients experience as light exertion, although health guidelines recommend at least moderate intensity when performing physical activities [36]. We decided to include sessions rated at least as light exertion (Borg 11) because we believed that incorporating light activities, such as walking and everyday activities, would better reflect the patients' everyday behaviour and actual physical activity patterns. This is also in line with current evidence, which recommends low-intensity exercise (RPE 11–13) for less trained individuals [37]. These two limitations preclude the possibility of analysing the total amount of physical activity and comparing the result with recommended health guidelines [36]. However, in patients with hip and knee osteoarthritis there is still limited evidence regarding the optimal intensity and dosage of exercise needed for clinical benefits [10,13] and it appears that low-intensity physical activity can benefit these patients [[4], [5], [6], [7]]. Furthermore, the aim of this study was to describe forms and types of physical activity that patients with hip or knee OA prefer and actually do. Our belief was that describing the frequency of physical activity sessions performed during the day and also including lighter exertion activities would better capture the patients' behaviour. This is also in accordance with OA guidelines, which advise patients to do as much as they can based on their OA symptoms [[10], [11], [12]]. Selection-bias may have occurred given that participants in the primary RCT had volunteered for a clinical trial on physical activity, and thus may have been more prone to perform physical activity than the average person with OA. Also, since they were informed that the aim was to evaluate different modes of physical activity interventions, the included participants could have been more likely to evaluate and report physical activity than the general population. Also, our inclusion of patients from a single region of Sweden and the fact that few men were included may be a limitation, since it affects the generalisation of our results to other populations. However, the population in this study can be assumed to be fairly representative of people with OA in the hip or knee.

## 5 Conclusions

Walking is the form of physical activity osteoarthritis patients most often choose, perform and maintain. Strength training appears to be infrequently practiced, and healthcare professionals could better support patients to find ways to incorporate this into their daily routines, instead of simply providing recommendations to engage in strength training as a stand-alone practice. Overall, supporting people to increase activities they already do (or used to do), and activities they are comfortable doing, may be a better strategy for maintaining physical activity in the long term.

## Author contributions

RB, AHE, MP and BS: Study conception and design together with ME, KB and LVK. RB: Did the analysis and interpretation of data and wrote the first draft together with AHE, MP and BS. RB, AHE, MP, BS, LK, ME and KB: Participated in the discussions and revisions of the manuscript and approved the final version.

## Role of the funding sources

The project received funding's from the Uppsala-Örebro Regional Research Council three times (RFR-81931, RFR-154901, RFR-213801) and from the Centre for Research and Development 10.13039/501100007051Uppsala University/Region Gävleborg two times (CFUG-158521, CFUG-572551). Financiers have not been part of the project design, collection, analysis or interpretation of data or in writing manuscript and not the decision to submit the manuscript for publication.

## Declaration of competing interest

The authors declare that they have no competing interest.
