# Supplementary material for: Walking is the form of physical activity people with osteoarthritis in the hip or knee choose and maintain after two years
Source: Osteoarthr Cartil Open. 2025 Sep 11;7(4):100681. doi: 10.1016/j.ocarto.2025.100681 (PMC12478240; doi:10.1016/j.ocarto.2025.100681)
Supplement: Multimedia component 1 [file mmc1.docx]

**Supplement 1.** Number of sessions in different forms of activities performed by 94 patients - at baseline and after one and two years.

| **Forms** | **Sessions baseline** | **Sessions 1 year** | **Sessions 2 year** |
| --- | --- | --- | --- |
| **Walking** | 221 | 264 | 233 |
| **Treadmill walking** | 8 | 10 | 8 |
| **Nordic walking** | 21 | 12 | 13 |
| **Housework** | 14 | 19 | 23 |
| **Gardening** | 21 | 27 | 29 |
| **Snow shovelling** | 2 | 8 | 6 |
| **Outdoor cycling** | 33 | 47 | 78 |
| **Indoor cycling** | 1 | 9 | 3 |
| **Gym training** | 3 | 6 | 19 |
| **Strength training** | 23 | 22 | 21 |
| **Swimming** | 1 | 2 | 2 |
| **Water-training** | 0 | 5 | 3 |
| **Yoga** | 0 | 4 | 4 |
| **Qigong** | 1 | 0 | 1 |
| **Jogging** | 1 | 1 | 0 |
| **Skiing** | 3 | 1 | 0 |
| **Skating** | 0 | 1 | 0 |
| **Dance** | 2 | 0 | 1 |
| **Zumba** | 1 | 6 | 4 |
| **Riding** | 1 | 0 | 1 |
| **Boule** | 1 | 1 | 1 |
| **Bowling** | 0 | 2 | 1 |
| **Curling** | 1 | 0 | 1 |
| **Total number of sessions** | 359 | 447 | 452 |
